# Electrophoresis of polar fluorescent tracers through the nerve sheath labels neuronal populations for anatomical and functional imaging

**DOI:** 10.1038/srep40433

**Published:** 2017-01-13

**Authors:** Matthew D. Isaacson, Berthold Hedwig

**Affiliations:** 1 Howard Hughes Medical Institute, Janelia Farm Research Campus, 19700 Helix Drive, Ashburn, VA 20147, USA; 2 Department of Zoology, University of Cambridge, Downing St, Cambridge, CB2 3EJ, UK.

## Abstract

The delivery of tracers into populations of neurons is essential to visualize their anatomy and analyze their function. In some model systems genetically-targeted expression of fluorescent proteins is the method of choice; however, these genetic tools are not available for most organisms and alternative labeling methods are very limited. Here we describe a new method for neuronal labelling by electrophoretic dye delivery from a suction electrode directly through the neuronal sheath of nerves and ganglia in insects. Polar tracer molecules were delivered into the locust auditory nerve without destroying its function, simultaneously staining peripheral sensory structures and central axonal projections. Local neuron populations could be labelled directly through the surface of the brain, and *in-vivo* optical imaging of sound-evoked activity was achieved through the electrophoretic delivery of calcium indicators. The method provides a new tool for studying how stimuli are processed in peripheral and central sensory pathways and is a significant advance for the study of nervous systems in non-model organisms.

For neuroanatomical studies and functional imaging the targeted delivery of dyes and indicators into neuron populations remains a fundamental challenge. In some animals the gene-targeted expression of fluorescent proteins in specific neuronal populations has become the dominant *in-vivo* labelling method^1^,^2^. In most experimental animals however, these genetic tools are not available. The classical technique for labelling the central or peripheral projection of neurons is the diffusion of dyes into cut nerves^3^,^4^. As this approach destroys the functional integrity of the nerves, simultaneous labelling in both directions is prevented and neuronal activity cannot be recorded. These shortcomings prompted us toward the development of an alternative dye delivery method that can be used in a variety of animals and maintains the integrity of the tissue. In this study we focused on the nervous system of locusts and crickets, which are widely used to study auditory processing^5^,^6^,^7^.

Inspired by methods of iontophoretic transdermal medication delivery^8^, in which an external electric field is used to deliver drugs through the skin, for anatomical and functional studies of insect auditory pathways we aimed to deliver tracers across the neural sheath, i.e. the neural lamella^9^ and the perineurium that form the outermost layer of connective tissue and glial cells covering nerves and ganglia. We initially focused on the locust auditory nerve, attaching the tip of a suction electrode (50 μm inner diameter) to the surface of the intact nerve halfway between the metathoracic ganglion and the hearing organ (Fig. 1a). The electrode was filled with the polar tracers Lucifer yellow or Texas Red-3,000 MW dextran. Whole-nerve field potentials and extracellular spike activity in response to acoustic stimuli indicated a good contact and tight seal between the electrode tip and the surface of the nerve. Pulsing current through the electrode (−40 μA, 250 ms pulse width at 1 Hz for 30 seconds) caused electrophoretic transfer of the dye from the pipette through the sheath into the auditory nerve (see Supplementary Fig. 1 for a diagram of the dye delivery apparatus). As the current pulses transiently and locally electroporated the sheath and the axonal membranes of the sensory neurons, these became permeable and the tracers were successfully delivered into the population of auditory afferents. Following the procedure, the specimens were kept at 4 °C and the dye allowed to spread for 24 hours. After dissection and standard histological processing, fluorescent imaging of the auditory organ and the CNS demonstrated the simultaneous anterograde and retrograde transport of the tracers. Both the peripheral cell bodies and dendrites of the scolopidial sensory neurons in the auditory organ and their central axonal projections in the auditory neuropils in the metathoracic ganglia were successfully stained. (Fig. 1b). With an increase in the total current injection time to 2 minutes, Cobalt ions as well as Alexa-568-10,000 MW dextran were also successfully delivered (Supplementary Fig. 2). With our protocol, dye spread to the mesothoracic ganglion 6 mm from the injection site, though dye concentrations in fibers ascending further became very weak. To investigate the ability of this technique to label neurons over even greater distances, we electrophoretically delivered Neurobiotin into the abdominal connective of the cricket CNS and allowed 2 days for the tracer to spread in both directions (Fig. 2a). After conventional antibody staining against Neurobiotin^10^ which served to enhance tracer detection, we observed numerous stained fibers reaching the entire length of the CNS (~20 mm), with the cell body and main dendrites of the cercal medial giant interneuron (MGI)^11^ clearly identifiable in the terminal ganglion as well as its putative axonal arborizations in the brain.

To explore further targets for the technique we attached the suction electrode to the surface of the locust brain, delivering Texas Red-3,000 MW dextran through the sheath into the dorso-lateral protocerebrum to which axons of the ascending auditory pathway project^12^ (Fig. 2b). The neural tissue directly beneath the injection site became highly saturated with dye, though fibers of ascending or descending interneurons and cell bodies of local brain neurons were clearly labelled outside this area, demonstrating that the technique will also allow tracing of neuron populations in ganglia and the brain. Next we aimed to adapt this labelling technique to allow optical imaging of neural activity through the delivery of calcium indicators. Current methods for the introduction of such indicators are mainly based on microelectrode electrophoresis^13^, bath application, or pressure-injection of cell membrane permeant indicators^14^,^15^. All these methods have limited ability to label large populations of neurons reliably. As our electrophoretic dye delivery imposed no observable physical damage to the nervous tissue, we targeted the intact locust auditory nerve ~1 mm to the metathoracic ganglion with the aim of labeling these sensory neurons for functional imaging in the central neuropils. Current injection times of 15 seconds were sufficient to deliver the calcium indicators Fluo-6 (Fig. 3) and Oregon Green (Supplementary Fig. 3) into the afferents; longer injection times overloaded the neurons with dye and reduced their viability. With this protocol the calcium indicators spread within 6 hours into the metathoracic auditory neuropils. Upon acoustic stimulation, activity of the live afferents as indicated by changes in the brightness of the fluorescence signal could be detected with a sensitive CCD camera. We presented 100 ms sound pulses in the range of 2–20 kHz at 70 dB SPL while imaging the neuronal responses. Normalizing the fluorescence signal to the pre-stimulus average, we observed a 5–40% raw ΔF/F correlated to the sound pulses in the 2–10 kHz frequency range. The optical signals accurately reflected the frequency-response characteristics of the electrical spike signals from the auditory nerve as determined by whole-nerve recordings taken prior to the dye-loading procedure (Fig. 3c), demonstrating that the method effectively labels auditory sensory neurons for functional imaging without apparently altering their encoding properties. Finally, we applied our method to the anterior ventral neuropil of the cricket brain where studies with intracellular recordings have identified a network of local auditory neurons for song pattern recognition^10^. Suction electrodes attached to the surface of this brain area reliably recorded auditory evoked field potentials which facilitated precise electrophoretic delivery of Fluo-6 through the sheath into the auditory neuropil, labelling numerous cell bodies and neurites (Fig. 4a). Presenting sound pulses at the carrier frequency of the species-specific calling song demonstrated an increase in relative fluorescence ΔF/F in auditory neurons, which formed a subset of all labelled neurons (Fig. 4b,c). Repeated presentation of the sound stimulus (0.1 s, 5 kHz, 70 dB SPL) revealed cell bodies of neurons close to the midline of the brain that were reliably activated by sound.

While the conditions of electrophoresis/electroporation used in this study successfully delivered dye for both anatomical and functional imaging, the parameter space was not completely explored and may further be improved to optimize dye delivery and minimize possible adverse effects. Higher current amplitudes (20–40 μA vs 5–10 μA) and extended injection times (1–4 minutes vs 15–30 seconds) increased the amount of dye delivered, though at the expense of disrupting signal transduction in the nerve as measured by recording whole-nerve activity before and after the procedure under the various conditions (Supplementary Fig. 4). Anatomical studies may not be concerned with signal disruption and opt for conditions that deliver more dye, while functional imaging experiments or behavioral studies that require unaffected neural function and lower indicator concentrations to prevent oversaturating the neuron with the indicator, can use very gentle conditions. Here only polar tracer molecules were used, though uncharged tracers might also be delivered by electro-osmosis as has been reported for transdermal iontophoresis^16^.

All functional imaging presented in this study was recorded about 6 hours after calcium indicator delivery, as this minimum time was required to allow a proper spreading of the dye. Neuronal activity in the neuropils could also be imaged 24 hours after dye delivery in the auditory nerve typically resulting in a lower ΔF/F, possibly due to the indicator not being retained within the neurons. Functional imaging may be possible beyond one day and even greater timescale after dye delivery, if the specimen can be kept in a good physiological condition to increase survival times.

Our non-invasive method delivers selectively anatomical and functional indicators into sensory pathways and neuropils of animals not amenable to genetic techniques; it thereby opens up a new range of imaging studies. To the best of our knowledge our method of dye delivery is currently the only one that allows simultaneous anterograde and retrograde labelling of fiber populations in insect peripheral nerves and that allows the labelling of local interneuron populations in fully-intact ganglia and brains. This confirms that this technique of electrophoretic dye loading can be successfully used for functional labelling of populations of neurons in various locations in the insect CNS. We propose that it may also be used in other invertebrate species and that it may advance functional imaging studies in non-genetic model systems. Since neurons could be successfully loaded with 10,000 MW polar dye molecules, we propose that DNA plasmids may also be capable of being delivered via a suction electrode, which so far has been demonstrated by local electroporation from a glass microelectrode^17^. Plasmids encoding fluorescent proteins for cell labelling would require longer survival times but would overcome the issue of local extracellular dye buildup at the injection site, as well as advance the possibility of genetic modification in more systems.

## Methods

### Animals and dissection

Locusts (*Schistocerca gregaria*) and crickets (*Gryllus bimaculatus*) were reared in crowded conditions (28 °C, 12:12 h light:dark cycles). Animals were selected for experiment 1–4 weeks following their final molt. The locust tympanal nerve and metathoracic ganglion were accessed by pinning the insect ventral side up in a lump of Plasticine and cutting a rectangular window in the ventral cuticle of the thorax. The dorsal side of the brain in locusts (for Fig. 2b) was exposed by cutting through the cuticle behind the eyes and bending the anterior portion of the head forward. The brain in female crickets (Fig. 4) was accessed by removing the cuticle between the eyes. In all cases some fat tissue and trachea were removed. For all experiments, insects were placed on a custom modified microscope stage that could be fitted beyond a dissecting microscope or into the fluorescence compound microscope used for imaging.

### Suction electrode procedures

Suction electrode pipettes were hand pulled to size from polypropylene tubing (CTPC500-1000-5; 0.5 mm ID, 1.0 mm OD, Paradigm Optics, Vancouver, WA, USA) over a heat source. Smooth ends were cut with a scalpel and inner tip diameters measured under a microscope. Pipette tips of ~50 μm ID were used for the locust auditory nerve and pipettes of ~100 μm ID were used for cricket connective stainings and for brain surface stainings. Pipettes were back-filled with a 6% tylose solution (Tylose H200 NP2, ShinEtsu, Wiesbaden, Germany) in saline, with tylose serving as a thickening agent to prevent the dye from diffusing along the shaft or leaking out of the tip. Dye solutions (2% dye in 4% tylose in water) were front-loaded into the pipette tip with suction. The pipette tip was inserted into a custom made electrode holder with an internal cavity filled with the tylose/saline solution - this cavity allowed for the formation of small air bubbles at the interface of the electrode and the current-carrying solution without them breaking the electrical connection to the pipette (Supplementary Fig. 1).

### Tracers used for anatomical and functional imaging

For anatomical studies, dye and tracer solutions used were Cobalt Chloride (Sigma-Aldrich 232696), Lucifer Yellow (Sigma-Aldrich L0259), Alexa Fluor 568-10,000 MW dextran, Texas Red-3,000 MW dextran (ThermoFisher Scientific D-22912 and D-3329), and Neurobiotin (Vector Laboratories SP-1120), were diluted to 2% in 4% tylose in water. For functional imaging, 2% solutions of Oregon Green 488 BAPTA-1 hexapotassium salt (ThermoFisher Scientific O-6806) and Fluo-6 hexapotassium salt (Luke Lavis, Jon Grimm, unpublished results) were used.

### Sound stimulation

Sound stimuli were generated using Cool Edit 2000 audio software (Syntrillium, Phoenix, AZ, USA, now Adobe Audition) and were presented by Sinus Live, Neo 13 s speakers (Conrad Electronics, Hirschau, Germany) placed 60 cm from the animal at 45 degrees left and right of the animals’ midline and calibrated to 70 dB SPL using a Brüel and Kjær measuring amplifier and microphone (models 2610 and 4939, respectively; Nærum, Denmark). Sound was not delivered in a sound-proof chamber so background noise (<50 dB) was present. For experiments studying frequency response characteristics of the target neurons (Fig. 3c), sound pulses were generated as pure tones of the following frequencies: 2, 4, 6, 8, 10, 15, and 20 kHz. For all other experiments, 5 kHz sound pulses were used. For whole nerve recordings, each sound pulse was 100 ms in duration and presented at 2 Hz (Fig. 3c, top; Supplementary Fig. 4), while for calcium imaging (Fig. 3c, bottom; Fig. 4c; Supplementary Fig. 3) each pulse was 200 ms in duration presented at 0.2 Hz to allow the calcium fluorescence signal to return to baseline between stimuli. For all experiments, each sound pulse began with a 10 ms linear rise to 70 dB and ended with a 10 ms linear fall.

### Whole-nerve recording and electrophoresis

**Figure 1:**
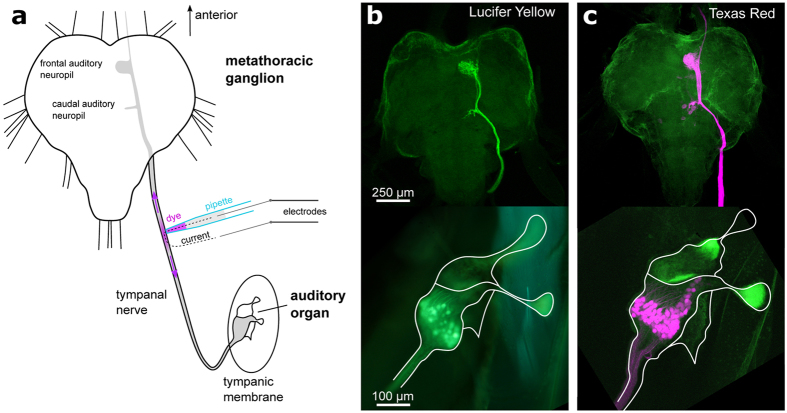
Neuroanatomical imaging of tracers delivered by electrophoresis across the nerve sheath. (**a**) Diagram of electrophoresis of dye across the sheath of the locust auditory nerve using a suction electrode. The auditory pathway in the ganglion is indicated in light grey. (**b**) Lucifer yellow and (**c**) Texas Red-3,000 MW dextran stainings of auditory afferents visualized by confocal microscopy of fixed/cleared metathoracic ganglia and auditory organ. Both the metathoracic ganglion and auditory organ were labelled in the same specimen by bidirectional spreading of the tracer. Background tissue autofluorescence and Lucifer yellow fluorescence is shown in green, Texas Red-3,000 MW dextran fluorescence in magenta.

**Figure 2:**
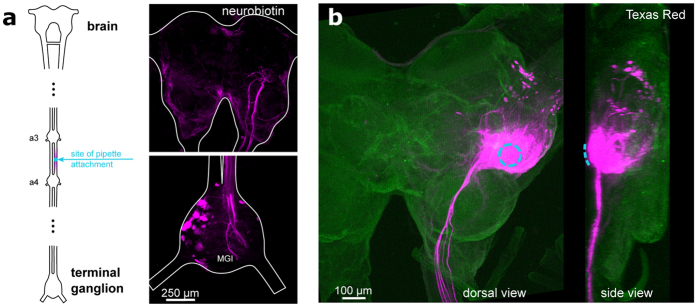
Long distance labelling and brain surface staining. (**a**) Delivery of Neurobiotin (neurobiotin-antibody staining shown in magenta) into a cricket connective between abdominal ganglia 3 and 4 labelled neuronal projections in the brain and terminal ganglion; the dendrites of the cercal Medial Giant Interneuron (MGI) are indicated. (**b**) Texas Red-3,000 MW dextran delivered into the locust brain through its surface without removing the sheath (teal dotted-line showing site of pipette attachment), imaged by confocal microscopy.

**Figure 3:**
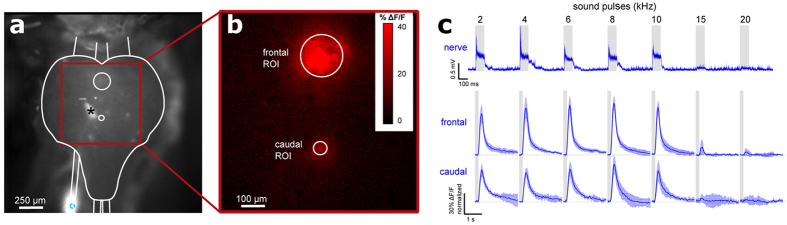
Functional imaging of metathoracic auditory neuropils by electrophoretic delivery of a calcium indicator in the locust auditory nerve. (**a**) Calcium indicator Fluo-6 delivered into the locust auditory nerve and imaged in the live metathoracic ganglion by epifluorescence microscopy. The bright white patches in the center-left of the ganglion (asterisk marking largest patch) are artefacts due to reflections in the saline. (**b**) Map of peak fluorescence change (ΔF/F) of the metathoracic ganglion in response to 5 kHz sound pulses; ROIs drawn for frontal and caudal neuropils. (**c**) Top, electrophysiological recording of tympanal nerve activity to sound pulses (indicated by light grey bars) of 2–20 kHz as measured by suction electrode prior to dye delivery; traces are rectified average signals (±s.e.m. in light blue, n = 4). Bottom, normalized ΔF/F traces showing frequency-response characteristics of frontal and caudal ROIs assigned to the corresponding auditory neuropils (±s.e.m., n = 6).

**Figure 4:**
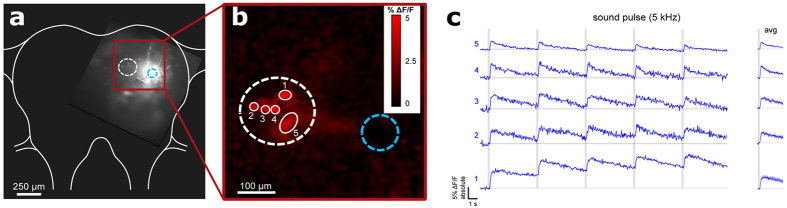
Sound-evoked activity directly visualized in the cricket brain. (**a**) Fluo-6 delivered into the anterior auditory neuropil in the cricket brain; site of pipette attachment circled in light blue and region of sound-evoked activity circled in white. (**b**) Fluorescence change map with ROIs drawn for active cell bodies or neuropil regions. (**c**) Absolute ΔF/F traces of each ROI showing activation correlated to 5 kHz sound pulses (indicated by light grey bars). Right, average ± s.e.m. of these 5 activations for each ROI.

After dye had been loaded into the suction electrode, the pipette tip was carefully sealed to the target tissue by gentle suction. A platinum reference electrode was placed in the hemolymph beside it. Whole-nerve activity was recorded via potential readings between the suction and reference electrode with a differential amplifier (model 1700, A-M Systems Inc. Carlsborg, WA, USA), and sampled at 44 kHz with Spike2 (v7, Cambridge Electronic Design, Cambridge, UK). For electrophoretic dye delivery, 40 μA current pulses with the polarity chosen to match that of the dye were delivered at 1 Hz and 250 ms width with a stimulus isolation unit (stimulus isolator A360, World Precision Instruments Ltd, Hitchin, UK) (Supplementary Fig. 1). Current pulses were delivered for 15 seconds for functional labelling and either 30 seconds or 2 minutes for anatomical stainings, depending on the tracer. Following current injection, the insect was kept at 4° for 6–48 hours to allow the dye to spread in the target neurons; the dye spreading speed was approximated at 500 μm/hr. Spread time for dye delivery into the locust tympanal nerve ~1 mm from the metathoracic ganglion and for delivery into brain tissue was 6 hours (Figs 2b, 3 and 4); for delivery in the tympanal nerve ~4 mm from the ganglion the spread time was 24 hours (Fig. 1b); and for delivery in the cricket A3-A4 connective the spread time was 48 hours to reveal projections in the brain and terminal ganglion (Fig. 2a).

### Anatomical and functional imaging

For anatomical imaging, after the dye had spread, ganglia and hearing organs were dissected, fixed, and cleared using standard histological methods with para-Formaldehyde, ethanol, and methyl salicylate. Cobalt stainings were first bathed in 2% ammonium sulfide for 5 minutes to produce a black cobalt-sulfide precipitate prior to fixation. Neurobiotin stainings were processed as previously described^10^. Images of stained tissue were taken with a Zeiss Axiophot microscope under bright-field or epifluorescent illumination using a Canon EOS 6D SLR camera. Confocal stacks of fluorescent stainings were obtained with a Leica SP5 with laser excitation light and filter cubes matching the excitation and emission spectra of the fluorophores. Max projections of confocal stacks were calculated in ImageJ (Abramoff *et al*. 2004) (Figs 1b and 2a,b).

For live imaging of delivered calcium indicators, insects tethered in plasticine were mounted under a Leica DMLFS microscope. The locust metathoracic ganglion was mechanically stabilized by cutting the ascending connectives, placed on a small silver plate and lifted away from surrounding tissues; the cricket brain was stabilized with minutien pins placed through the head cuticle. The microscope was fitted with a monochromator (Cairn, Faversham, UK; 488 nm, 10 nm band width) for indicator excitation and a cooled CCD camera (Andor iXon DV887, Andor Technologies, Belfast, UK) for image recording. Camera image timing was controlled by an external trigger set at a rate of 20 Hz and recorded simultaneously with an acoustic stimulus using Spike2. Image time-series’ were motion-stabilized by template matching in ImageJ. ΔF/F fraction change in fluorescence maps were calculated by normalizing the measured fluorescence of all frames to the average of the first 50 frames pre-stimulus, selecting the frame with the peak response, and smoothing the frame with the “Mean 3D” filter in ImageJ by 2 pixels in the x, y, and t dimensions (Figs 3b and 4b, Supplementary Fig. 3).

### Data analysis

During electrophysiological recordings of the locust tympanal nerve, animals were presented with numerous repetitions of each sound pulse: 13–25 repetitions for each frequency in Fig. 2c (top) and 25–50 repetitions for the 5 kHz pulses delivered for Supplementary Fig. 4. A rectified average recording for each individual and condition was calculated by taking the root mean square of the multiple repetitions at each data point; the signal for each repetition being aligned to the stimulus onset. The recordings displayed were down-sampled to 4.4 kHz by averaging in 10-sample bins. An overall average/SEM signal was calculated from the rectified average recordings from 4 insects.

In functional imaging experiments, ΔF/F traces for drawn ROIs were calculated for each ROI by the per-frame average fluorescence change relative to the pre-stimulus average (e.g. Fig. 4c, left). An average ΔF/F trace for each staining and condition was calculated by averaging the ΔF/F traces for 5 repetitions of the presented stimulus in that staining (Fig. 4c, right). In order to calculate the average ΔF/F trace over multiple stainings (Fig. 3c, middle and bottom), the average trace for each staining was first normalized to its own maximal response since the absolute response varied significantly between stainings, from 5–40% maximum ΔF/F. The average and SEM was then calculated from the normalized average traces from the multiple stainings.

## Additional Information

**How to cite this article**: Isaacson, M. D. and Hedwig, B. Electrophoresis of polar fluorescent tracers through the nerve sheath labels neuronal populations for anatomical and functional imaging. *Sci. Rep.* **7**, 40433; doi: 10.1038/srep40433 (2017).

**Publisher's note:** Springer Nature remains neutral with regard to jurisdictional claims in published maps and institutional affiliations.

## Supplementary Material

Supplementary Information
