# Supplementary material for: Electrophoresis of polar fluorescent tracers through the nerve sheath labels neuronal populations for anatomical and functional imaging
Source: Sci Rep. 2017 Jan 13;7:40433. doi: 10.1038/srep40433 (PMC5233955; doi:10.1038/srep40433)
Supplement: Supplementary Information [file srep40433-s1.pdf]

Electrophoresis of polar fluorescent tracers through the nerve sheath labels neuronal populations for anatomical and functional imaging

Matthew D. Isaacson and Berthold Hedwig

Supplementary Information

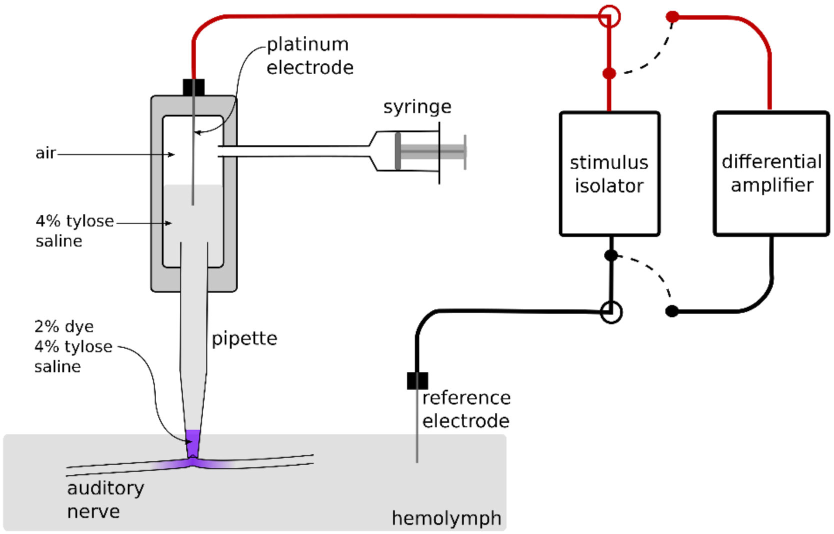

Supplementary Figure 1: **Diagram of apparatus for dye delivery and whole-nerve recording.** A custom designed electrode holder connects a pipette front-loaded with dye solution to a syringe for creating suction and to a platinum electrode for measuring voltage or supplying current. The target nerve of an insect is attached to the pipette tip with gentle suction. A reference electrode in the hemolymph surrounding the target nerve closes the electrical connection to the pipette electrode. The electrodes can be connected either to a differential amplifier for whole-nerve recording or to a stimulus isolator unit for applying direct current for electrophoretic dye delivery.

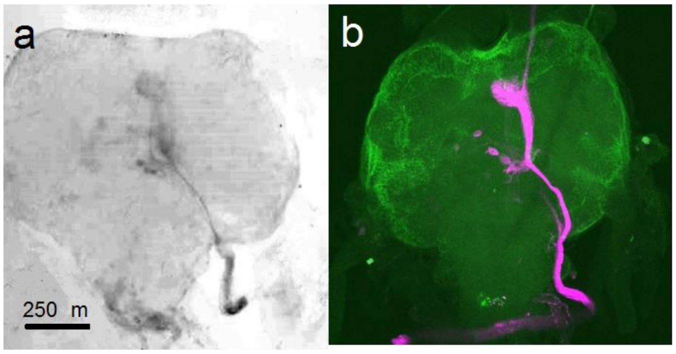

Supplementary Figure 2: **Anatomical imaging of locust auditory neuropils using other molecular tracers.** (**a**) Bright-field imaging of cobalt-sulfide precipitate in locust auditory neuropils following electrophoretic delivery of cobalt into the auditory nerve. **(b)** Epifluorescence imaging of auditory neuropils following electrophoretic delivery of Alexa 568-10,000 MW dextran.

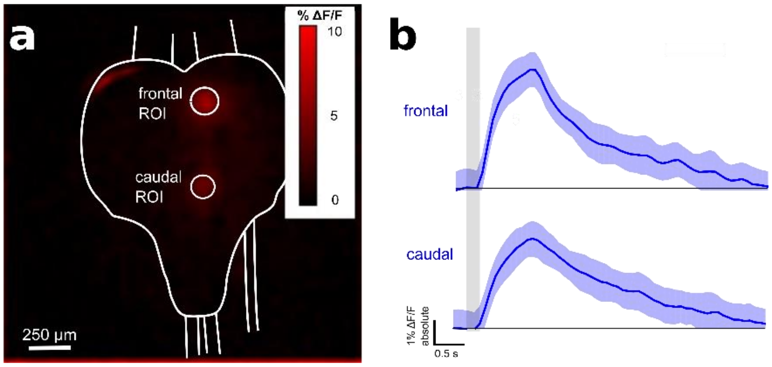

Supplementary Figure 3: **Functional imaging in locust auditory neuropils using Oregon Green 488. (a)** Fluorescence change map of locust metathoracic ganglion during acoustic stimulation following electrophoretic delivery of Oregon Green 488 BAPTA-1. ROIs drawn for frontal and caudal neuropils **(b)** Average fluorescence change of auditory neuropils during 20 stimulations in a single locust staining (± s.e.m. in light blue).

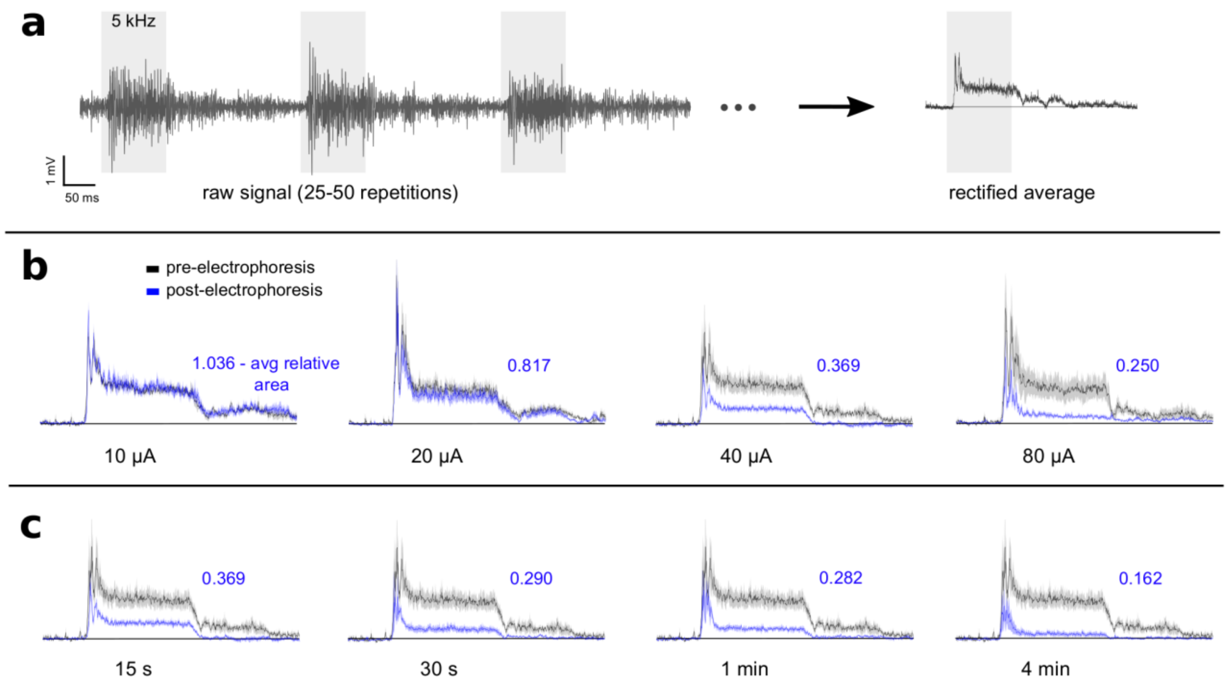

Supplementary Figure 4: **Effects of electrophoretic parameters on sound-evoked auditory nerve potentials.** (**a**) Illustration of calculating the rectified average signals from multiple repetitions of the 5 kHz stimulus-evoked raw whole nerve field potentials. Due to background noise being present while delivering the sound stimuli, a minimum of 25 repetitions of sound-evoked activity were averaged for each recording. (**b**) Effects of increasing the electrophoresis current amplitude on sound-evoked potentials: Current pulses of 10, 20, 40, and 80 µA were delivered for 15 seconds to the auditory nerve of different sets of locusts. Pre-electrophoresis signals were recorded prior to any current injection; post-electrophoresis signals were recorded immediately after. The area under the curve of the post-electrophoresis responses relative to their corresponding pre-electrophoresis recordings are displayed for each condition. (**c**) Effects of increasing current injection duration on sound-evoked potentials: After a pre-electrophoresis recording was taken, a total of 4 minutes of 40 µA current injection was delivered, with additional recordings being taken at intervals of 15 s, 30 s, 1 min, and 4 min. Current injection for all conditions shown were delivered with a 250 ms pulse at 1 Hz, with Fluo-6 loaded in the pipette.
